# Education Research: Evaluating Racial, Ethnic, and Gender Diversity Trends in Neurology Residency Programs Between 2007 and 2020

**DOI:** 10.1212/NE9.0000000000200264

**Published:** 2025-10-24

**Authors:** George N. Umegboh, Shani A. Williams, Anne-Emilie J. Rouffiac, Ethan Register, Saba S. Paracha, Sanaya Daruvala, Bethany J. Gentilesco, Elijah M. Persad-Paisley, Neishay Ayub

**Affiliations:** 1 The Warren Alpert School of Medicine of Brown University, Providence, RI;; 2 Department of Neurology, Rhode Island Hospital, The Warren Alpert Medical School of Brown University, Providence, RI; and; 3 Department of Internal Medicine, Rhode Island Hospital, The Warren Alpert Medical School of Brown University, Providence, RI.

## Abstract

**Background and Objectives:**

With increasing efforts to ensure a racially and ethnically diverse physician workforce, it is unclear whether the improved diversity changes in medical schools have been reflected among neurology residents. We aimed to examine the demographic changes of neurology residency applicants and matriculants relative to their medical school graduate counterparts.

**Methods:**

This was a population-based repeated cross-sectional study of Association of American Medical Colleges data on self-reported race, ethnicity, and gender of US medical school graduates, neurology residency applicants, and neurology residency matriculants from 2007 to 2020. The representation quotient (RQ) for each applicant and matriculant cohort was calculated from the ratio of the percentage of a racial, ethnic, and gender group in the population to that of those graduating medical school. Median RQs greater than 1 indicated that a cohort is overrepresented compared with their medical school graduate counterparts, whereas RQs less than 1 indicated underrepresentation. Mann-Whitney *U* tests were used to assess statistical changes in representation between applicants and matriculants within each racial and gender identity. Yearly changes in RQ were assessed using linear regressions for each race by gender.

**Results:**

Asian men (RQ_app_ = 1.20 [interquartile range (IQR) 1.13–1.28]), Black men (RQ_app_ = 1.23 [IQR 0.94–1.39]), and Hispanic men (RQ_app_ = 1.16 [IQR 0.89–1.29]) were overrepresented as applicants. RQ_app_ for Asian men remained stagnant while all other applicants trended toward increased representation. Hispanic men were the only group to have a significant increase in RQ_mat_ during the study period. Asian women (RQ_app_ = 0.98 vs RQ_mat_ = 1.12; *p* = 0.006) and White women (RQ_app_ = 0.40 vs RQ_mat_ = 0.79; *p* < 0.001) experienced increases in representation when transitioning to matriculants. Black men (RQ_app_ = 1.23 vs RQ_mat_ = 0.64; *p* < 0.001) experienced the largest reduction in representation, when comparing applicants with matriculants, among all groups. Black women (RQ_app_ = 0.58 vs RQ_mat_ = 0.59; *p* = 0.49) were underrepresented throughout application.

**Discussion:**

Racially minoritized women were underrepresented among neurology applicants, and most did not show significant increases in representation as matriculants. Black men experienced the largest magnitude reduction in RQ when transitioning from applicants to matriculants. There is a need for greater professional support for applicants from historically underrepresented backgrounds to ensure their equitable recruitment into neurology.

## Introduction

Studies have found that the supply of neurologists does not match the demand of an aging US population with increased need for neurologic care.^1^ A 2013 Workforce Task Force report from HIS Healthcare and Pharma predicted a national neurologist shortfall of 19% by 2025, an increase from 11% in 2012.^1,2^ The implications of this workforce shortage were illustrated in a 2020 analysis of health care access for Medicare patients with neurologic conditions.^3^ This study found that 20.6% of patients in rural areas were seen by a neurologist compared with 27% of those who lived in urban areas, despite similar prevalence in neurologic conditions across regions.^3^ Furthermore, the racial composition of the United States has also shifted dramatically in recent decades, with racial and ethnic minorities projected to represent more than half of the population by 2050.^4^ This projection has not been reflected among the physician workforce, a historically homogeneous population that must paradoxically deliver health care to an increasingly diverse patient cohort.

Patients routinely experience markedly different health outcomes that vary with racial background.^5,6^ These disparities occur at various points in health care, from timely referrals by primary care providers to disease management in the inpatient and outpatient setting.^1^ Compared with their White counterparts, racial minority neurology patients are less likely to be seen by a neurologist. They are less likely to receive acute care, such as tissue plasminogen activator or mechanical thrombectomy after stroke, and are less likely to receive appropriate clinical treatment for other chronic neurologic conditions, such as Parkinson disease and epilepsy.^7-11^

Increasing the representation of neurology physicians who identify as Black, Indigenous, and People of Color is unlikely to overcome highly prevalent health disparities on its own. However, inclusion of groups who have been historically underrepresented in medicine (URiM) has been identified as a potential solution to improve health outcomes for minoritized patients.^12-15^ As a result, US medical schools have implemented initiatives to increase the number of qualified medical student matriculants to their institutions to mitigate health care disparities.^16^ These efforts have contributed to a significant increase in the racial and gender diversity within medical student populations since the 2000s.^17,18^ However, it is not clear whether these changes have been reflected in neurology.

As of 2019, the percentage of self-identified women (43%), African American (3.7%), and Asian/Pacific Islander (20%) neurology residents statistically increased from that of 2011 while the percent representation of Hispanic (5.1%) individuals remained unchanged.^19^ There is limited information regarding the racial and gender trends of candidates entering neurology residency, specifically at the stage of application and matriculation. Those studies that examine diversity in the neurology workforce focus on the total counts and percentages of different identities in neurology residency alone.^20^ Without a reference point, it is difficult to compare changes in absolute representation between groups of varying sizes. This comparison is especially challenging to evaluate in the application pathway because the applicant pool decreases in size from applicant to matriculant status. An ideal measure of inequity must, therefore, be independent of population size and the unit of measurement used.^21^ Representation quotients (RQs) have been identified as a measure of inequity that satisfies these requirements.^22-24^ The purpose of this study was to, therefore, implement RQ methodology to evaluate the racial and gender representation of neurology residency applicants and matriculants by using medical school graduating classes as reference populations.

## Methods

### Data Acquisition

Annual reports on allopathic and osteopathic US medical school graduates (USMGs), neurology residency applicants, and neurology residency matriculants were obtained from the Association of American Medical Colleges (AAMC) for all academic years between and including 2007–2008 and 2019–2020 on request. Matriculants in this study represent individuals who applied to and enrolled in a neurology residency program during the 13-year study period. Gender, as defined by the AAMC, represents an individual's self-reported identity and not necessarily their biological sex assigned at birth. Genders included men and women. The races and ethnicities included American Indian/American Native (hereinafter referred to as “AI/AN”); Asian; Black or African American (hereinafter referred to as “Black”); Hispanic, Latino, or of Spanish Origin (hereinafter referred to as “Hispanic”); Native Hawaiian or Other Pacific Islander (hereinafter referred to as “NHoPI”); and White. AI/AN or NHoPI groups were not reported in the Results section because of low absolute counts and increased variance in statistical analysis. Individuals who self-identified as “other,” “unknown race/ethnicity,” or “non-US citizen and nonpermanent resident” did not specify a race or ethnicity. The authors did not deem it appropriate to evaluate the representation of these individuals under an assumed shared racial or ethnic identity. Therefore, these individuals were excluded from the statistical analysis for this study. Data for the “total unduplicated men” and “total unduplicated women” cohorts included counts from these excluded groups and were consequently not referenced in the Results section.

Table 1 lists all individuals to accurately demonstrate the demographic data of all groups in this study cohort. Between the academic years of 2002–2003 and 2012–2013, the AAMC asked respondents about their racial and ethnic background in 2 separate questions. After the 2012–2013 academic year, the AAMC has collected this information in a single question, allowing individuals to select multiple races and ethnicities. Before 2020, AAMC residency application data only included racial and ethnic background information for individuals who self-identified as US citizens or permanent residents. The authors did not deem it necessary to manipulate the data any differently than it was originally presented for the purposes of analysis; this is consistent with the approaches used in previous studies that use similar data sets.^23,24^

**Table 1:** Racial, Ethnic, and Gender Demographics of US Allopathic Medical School Graduates and US Neurology Residency Applicants/Matriculants From 2007 to 2020

Race, ethnicity, and gender identity	Medical school graduates (%)^a^	Neurology residency applicants (%)^a^	Neurology residency matriculants (%)^a^
AI/AN men	455 (0.18)	67 (0.19)	24 (0.22)
AI/AN women	469 (0.18)	27 (0.08)	16 (0.15)
Asian men	26,718 (10.39)	4,278 (12.40)	1,180 (10.92)
Asian women	27,429 (10.67)	3,483 (10.09)	1,253 (11.60)
Black men	5,833 (2.27)	946 (2.74)	168 (1.56)
Black women	10,046 (3.91)	701 (2.03)	241 (2.23)
Hispanic men	7,682 (2.99)	1,119 (3.24)	355 (3.29)
Hispanic women	7,664 (2.98)	840 (2.43)	336 (3.11)
NHoPI men	154 (0.06)	17 (0.05)	8 (0.07)
NHoPI women	136 (0.05)	16 (0.05)	10 (0.09)
White men	81,567 (31.72)	5,372 (15.57)	2,716 (25.14)
White women	68,622 (26.69)	3,732 (10.82)	2,240 (20.73)
Other race/ethnicity men	2,018 (0.78)	543 (1.57)	197 (1.82)
Other race/ethnicity women	1,681 (0.65)	330 (0.96)	152 (1.41)
Unknown race/ethnicity men	1,097 (0.43)	999 (2.90)	20 (0.19)
Unknown race/ethnicity women	840 (0.33)	791 (2.29)	13 (0.12)
Non-US citizen men	2,050 (0.80)	7,610 (22.05)	1,495 (13.84)
Non-US citizen women	2,021 (0.79)	4,232 (12.26)	1,052 (9.74)
Total unduplicated men	133,062 (51.75)	20,584 (59.65)	5,811 (53.79)
Total unduplicated women	124,077 (48.25)	13,923 (40.35)	4,989 (46.18)
Total unduplicated individuals	257,139	37,079	10,803

Abbreviations: AI/AN = American Indian/Alaska Native; Hispanic = Hispanic, Latino, or of Spanish Origin; NHoPI = Native Hawaiian or Other Pacific Islander.

a Individuals may select more than 1 race/ethnicity. As such, percentages for these groups may not add up to 100%.

### RQ Calculations and Statistical Analysis

The method for standardizing applicant and matriculant representation for each racial, ethnic, and gender group was adapted from prior research.^23,24^ The RQs for neurology residency applicants (RQ_app_) and matriculants (RQ_mat_) were calculated by dividing the percentage of each racial, ethnic, or gender identity in a given cohort by the percentage of that same identity among US medical school graduating classes in the same year. RQs greater than 1 suggest that applicants or matriculants of a subgroup are overrepresented compared with their medical school graduate counterparts; RQs less than 1 indicate underrepresentation. Mann-Whitney *U* tests were used to evaluate statistical differences between the median RQ_app_ and RQ_mat_ within subgroups. Mann-Whitney *U* tests were 2-tailed, and *p* values less than 0.05 were deemed significant. Trends in applicant and matriculant representation were examined using linear regression models with RQ and academic year as the dependent and independent variables, respectively. *p* Values less than 0.05 for individual regressions were deemed significant. Multiple simple tests were completed for each subgroup to evaluate for statistical significance. The result of each individual test was important to this study and, as such, the Bonferroni correction was not required for data analysis.^25^ Median RQs and interquartile ranges (IQRs) are reported unless otherwise noted. Statistical analyses were performed using R statistical software.^26^ The authors adhered to the Strengthening the Reporting of Observational Studies in Epidemiology reporting guidelines.^27^

### Standard Protocol Approvals, Registrations, and Participant Consents

This study was reviewed by the local IRB and was deemed to be exempt because of its designation as “research—not human subjects research.”

### Data Availability

Anonymized data not published within this article can be made available on reasonable request from any qualified investigator.

## Results

### Population Demographics

The total number of medical school graduates and neurology residency applicants and matriculants during the study period is summarized in Table 1. The median RQ values of neurology residency applicants and matriculants for the study cohorts are summarized in Table 2. Among applicants, Asian men (RQ_app_ = 1.20 [IQR 1.13–1.28]), Black men (RQ_app_ = 1.23 [IQR 0.94–1.39]), and Hispanic men (RQ_app_ = 1.16 [IQR 0.89–1.29]) were the only groups overrepresented during the study period. Asian men (RQ_mat_ = 1.05 [IQR 0.95–1.14]), Asian women (RQ_mat_ = 1.12 [IQR 0.99–1.19]), Hispanic men (RQ_mat_ = 1.15 [IQR 0.88–1.20]), and Hispanic women (RQ_mat_ = 1.02 [IQR 0.98–1.16]) were overrepresented at the matriculant stage. All other identities were underrepresented as neurology residency matriculants.

**Table 2:** Median RQ Values and RQ Trend Over Time for Neurology Applicants and Residents From 2007 to 2020

Race, ethnicity, and gender identity	Median RQ value (IQR)	RQ slope (95% CI)	RQ slope *p* value
Neurology residency applicants			
AI/AN men	2.04 (0.39–4.56)	2.53 × 10^−1^ (−1.03 × 10^−1^ to 6.09 × 10^−1^)	0.15
AI/AN women	0.41 (0.16–1.40)	8.10 × 10^−2^ (−5.58 × 10^−2^ to 2.18 × 10^−1^)	0.22
AI/AN men and women	1.46 (0.29–2.59)	1.67 × 10^−1^ (−2.82 × 10^−2^ to 3.62 × 10^−1^)	0.09
Asian men	1.20 (1.13–1.28)	4.46 × 10^−4^ (−1.57 × 10^−2^ to 1.66 × 10^−2^)	0.95
Asian women	0.98 (0.85–1.01)	1.85 × 10^−2^ (4.87 × 10^−3^ to 3.21 × 10^−2^)	0.01^a^
Asian men and women	1.06 (1.03–1.16)	9.65 × 10^−3^ (−4.55 × 10^−3^ to 2.39 × 10^−2^)	0.16
Black men	1.23 (0.94–1.39)	5.29 × 10^−2^ (9.61 × 10^−3^ to 9.61 × 10^−2^)	0.02^a^
Black women	0.58 (0.40–0.61)	2.60 × 10^−2^ (1.57 × 10^−2^ to 3.64 × 10^−2^)	<0.001^a^
Black men and women	0.82 (0.60–0.92)	3.86 × 10^−2^ (2.09 × 10^−2^ to 5.62 × 10^−2^)	<0.001^a^
Hispanic men	1.16 (0.89–1.29)	4.13 × 10^−2^ (1.91 × 10^−2^ to 6.34 × 10^−2^)	0.002^a^
Hispanic women	0.91 (0.61–1.02)	5.06 × 10^−2^ (3.08 × 10^−2^ to 7.03 × 10^−2^)	<0.001^a^
Hispanic men and women	1.08 (0.75–1.14)	4.61 × 10^−2^ (2.73 × 10^−2^ to 6.48 × 10^−2^)	<0.001^a^
NHoPI men	0.52 (0.05–1.14)	1.01 × 10^−1^ (−4.67 × 10^−1^ to 6.69 × 10^−1^)	0.71
NHoPI women	0.73 (0.00–2.05)	2.48 × 10^−1^ (5.35 × 10^−3^ to 4.91 × 10^−1^)	0.05
NHoPI men and women	0.65 (0.23–1.94)	1.76 × 10^−1^ (−1.10 × 10^−1^ to 4.62 × 10^−1^)	0.20
White men	0.50 (0.40–0.55)	2.08 × 10^−2^ (1.76 × 10^−2^ to 2.40 × 10^−2^)	<0.001^a^
White women	0.40 (0.35–0.46)	1.25 × 10^−2^ (7.99 × 10^−3^ to 1.71 × 10^−2^)	<0.001^a^
White men and women	0.46 (0.38–0.51)	1.69 × 10^−2^ (1.41 × 10^−2^ to 1.98 × 10^−2^)	<0.001^a^
Total unduplicated men	1.16 (1.11–1.18)	−7.81 × 10^−3^ (−1.13 × 10^−3^ to −4.27 × 10^−3^)	<0.001^a^
Total unduplicated women	0.83 (0.80–0.87)	8.45 × 10^−3^ (5.29 × 10^−3^ to 1.16 × 10^−3^)	<0.001^a^
Neurology residency matriculants			
AI/AN men	2.36 (0.39–3.12)	3.42 × 10^−1^ (7.12 × 10^−2^ to 6.14 × 10^−1^)	0.02^a^
AI/AN women	0.97 (0.37–2.18)	1.91 × 10^−1^ (1.54 × 10^−2^ to 3.66 × 10^−1^)	0.04^a^
AI/AN men and women	2.30 (0.41–2.81)	2.58 × 10^−1^ (1.22 × 10^−1^ to 3.94 × 10^−1^)	0.001^a^
Asian men	1.05 (0.95–1.14)	−1.43 × 10^−2^ (−3.12 × 10^−2^ to −2.52 × 10^−3^)	0.09
Asian women	1.12 (0.99–1.19)	−1.36 × 10^−2^ (−2.83 × 10^−2^ to 1.09 × 10^−3^)	0.07
Asian men and women	1.10 (1.00–1.16)	−1.38 × 10^−2^ (−2.48 × 10^−2^ to −2.71 × 10^−3^)	0.02^a^
Black men	0.64 (0.54–0.81)	1.12 × 10^−2^ (−1.56 × 10^−3^ to 3.79 × 10^−2^)	0.38
Black women	0.59 (0.49–0.64)	8.34 × 10^−3^ (−1.71 × 10^−2^ to 3.38 × 10^−2^)	0.49
Black men and women	0.59 (0.57–0.70)	1.06 × 10^−2^ (−4.95 × 10^−3^ to 2.61 × 10^−2^)	0.16
Hispanic men	1.15 (0.88–1.20)	3.97 × 10^−2^ (3.29 × 10^−3^ to 7.61 × 10^−2^)	0.04^a^
Hispanic women	1.02 (0.98–1.16)	3.20 × 10^−2^ (−5.54 × 10^−3^ to 6.95 × 10^−2^)	0.09
Hispanic men and women	1.13 (0.95–1.24)	3.61 × 10^−2^ (8.95 × 10^−3^ to 6.33 × 10^−2^)	0.01^a^
NHoPI men	0.00 (0.00–2.55)	7.53 × 10^−2^ (−5.62 × 10^−1^ to 7.12 × 10^−1^)	0.80
NHoPI women	1.25 (0.00–3.55)	−1.87 × 10^−1^ (−6.37 × 10^−1^ to 2.64 × 10^−1^)	0.38
NHoPI men and women	1.56 (0.00–3.59)	−5.47 × 10^−2^ (−5.19 × 10^−1^ to 4.09 × 10^−1^)	0.80
White men	0.81 (0.76–0.85)	7.35 × 10^−3^ (−1.38 × 10^−3^ to 1.61 × 10^−2^)	0.09
White women	0.79 (0.76–0.81)	1.53 × 10^−3^ (−6.41 × 10^−3^ to 9.46 × 10^−3^)	0.68
White men and women	0.80 (0.77–0.82)	4.62 × 10^−3^ (−1.39 × 10^−3^ to 1.06 × 10^−2^)	0.12
Total unduplicated men	1.04 (1.02–1.06)	−2.40 × 10^−3^ (−6.67 × 10^−3^ to 1.87 × 10^−3^)	0.24
Total unduplicated women	0.96 (0.93–0.98)	2.53 × 10^−3^ (−2.04 × 10^−3^ to 7.09 × 10^−3^)	0.25

Abbreviations: AI/AN = American Indian/Alaska Native; IQR = interquartile range; NHoPI = Native Hawaiian or Other Pacific Islander; RQ = representation quotient.

a Statistically significant at *p* < 0.05.

### Trends in Representation

**Figure 1:**
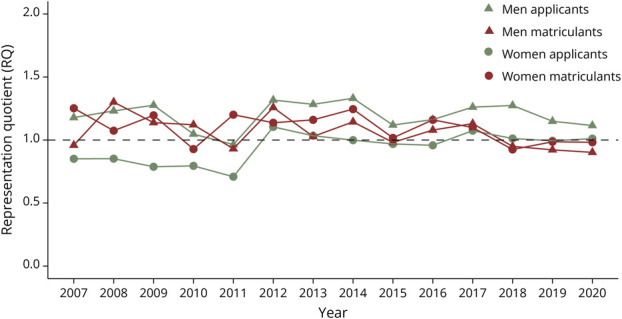
Asian Neurology Applicants and Matriculants Yearly trends in representation quotients (RQs) by race, ethnicity, and gender of Asian US neurology residency applicants/matriculants from 2007 to 2020. Dashed line: RQ = 1.

**Figure 2:**
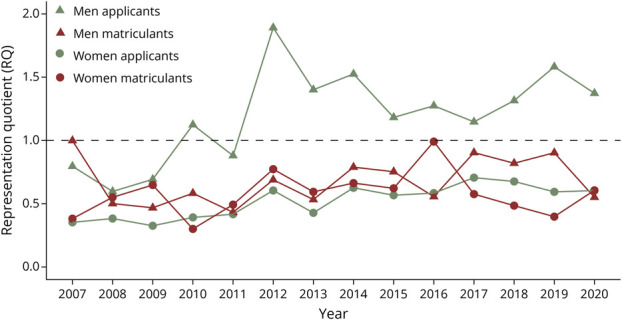
Black Neurology Applicants and Matriculants Yearly trends in representation quotients (RQs) by race, ethnicity, and gender of Black US neurology residency applicants/matriculants from 2007 to 2020. Dashed line: RQ = 1.

**Figure 3:**
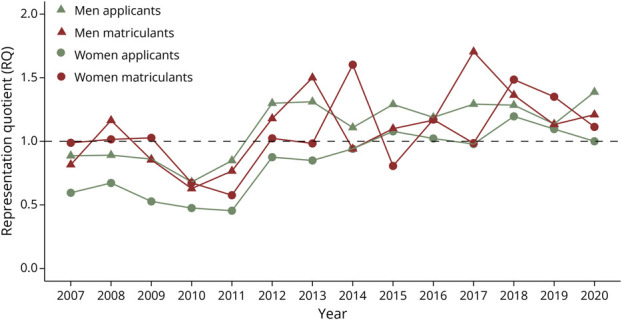
Hispanic Neurology Applicants and Matriculants Yearly trends in representation quotients (RQs) by race, ethnicity, and gender of Hispanic US neurology residency applicants/matriculants from 2007 to 2020. Dashed line: RQ = 1.

**Figure 4:**
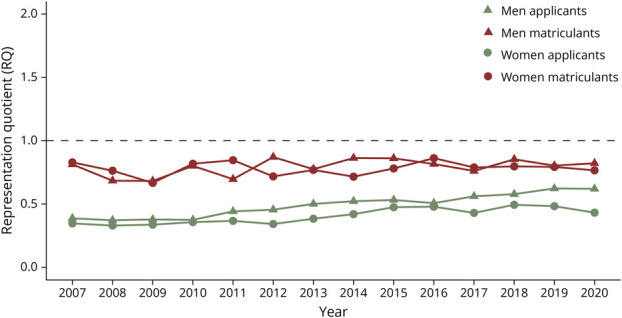
White Neurology Applicants and Matriculants Yearly trends in representation quotients (RQ) by race, ethnicity, and gender of White US neurology residency applicants/matriculants from 2007 to 2020. Dashed line: RQ = 1.

The results from the linear regressions highlighting trends in RQ between 2007 and 2020 are summarized in Table 2. Among applicants, Asian men had no significant change in representation while all other groups experienced increases in RQ_app_ over the study period (Figure 1). Asian matriculants were the only group to have statistically significant decreases in RQ_mat_ between 2007 and 2020 (Figure 1). Hispanic men were the only group to experience a significant increase in RQ_mat_ during this time (Figure 3). The representation of all other matriculant groups remained stagnant or decreased during the study period (Figures 1–4).

Only Asian men (*p* = 0.008) and Black men (*p* < 0.001) experienced statistically significant decreases in representation when comparing applicants with matriculants (Table 2). White men (*p* < 0.001), White women (*p* < 0.001), and Asian women (*p* = 0.006) gained representation when transitioning from neurology applicants to matriculants (Table 2). Black women (*p* = 0.49), Hispanic women (*p* = 0.06), and Hispanic men (*p* = 0.73) did not have substantial changes in representation between applicants and matriculants. White women (RQ_app_ = 0.40 vs RQ_mat_ = 0.79; *p* < 0.001) remained underrepresented as matriculants despite a 97.5% increase in representation from the applicant stage (Table 2). Black men (RQ_app_ = 1.23 vs RQ_mat_ = 0.64; *p* < 0.001) experienced the largest decrease in representation among all groups with a 48% reduction from the applicant to matriculant stage (Table 2).

## Discussion

The purpose of this study was to systematically evaluate racial, ethnic, and gender representation among incoming neurology residents compared with that of medical school graduating classes. We ultimately found that the representation of Black men and women matriculating into neurology residency programs has not matched that of those graduating medical schools from 2007 to 2020. Black women were among the least represented groups at both stages and experienced statistically stagnant representation over time among matriculants. On the contrary, Black men and Asian men were 2 of the most represented groups in the applicant cohort but experienced significant decreases in representation from applicant to matriculant status. Furthermore, Black men were the only group to have a significant shift from overrepresentation as applicants to underrepresentation as matriculants. The use of RQs—which represent normalized cohort proportions—further highlights the degree to which historically excluded groups continue to be underrepresented in the neurology workforce.

There is limited information regarding representation trends of different racial and gender identities entering the neurologic workforce. One study found that White neurology residents were the most overrepresented group while Hispanic and Black neurology residents were considerably underrepresented between 2011 and 2018.^20^ Our findings regarding the underrepresentation of Black individuals are consistent with those of their study. However, when considering neurology residency demographics in the context of those in medical school, we found that self-identified White individuals were also underrepresented as applicants and matriculants.

On further evaluation, White cohorts experienced a statistically significant increase in representation as matriculants when compared with applicant representation. Asian women experienced a significant shift from being underrepresented as applicants to overrepresentation in the matriculant group. However, most URiM cohorts did not experience a similar shift in representation. URiM applicant cohorts generally demonstrated gradual improvement in underrepresentation, but most matriculant groups did not experience significant increase in representation over the 13-year study period. In fact, Asian men and women were nearly equally represented as neurology applicants compared with their medical school graduate counterparts yet experienced a significantly negative trend in representation as neurology matriculants.

The reason for the observed discrepancies in applicant and matriculant representation is not immediately apparent. “Neurophobia”—a well-recognized fear of neurology among medical students—may contribute to underrepresentation in applicants.^28,29^ This aversion to pursuing neurology develops over the course of medical training with factors such as unfavorable early neuroscience experiences and finances playing a significant role.^30-32^ Neurophobia alone does not explain the discrepancy in matriculant representation seen among URiM groups, but it does provide meaningful insight into the challenges that make neurology less appealing to potential applicants.

Barriers to professional development during medical school may also contribute to the low representation seen among neurology residency applicants and matriculants. URiM trainees often face sociohistorical barriers, which can hinder personal and professional development.^33,34^ One study found a significant association between racial/ethnic discrimination and personal/professional identity formation (PPIF) among US medical students.^35^ Within this national cohort, Black students and URiM students disproportionately experienced discrimination and reported lower levels of PPIF during medical school. Factors including stereotype threat and sociocultural context may further influence professional development in various medical fields.^33,34,36^

Aspects including matching into a different residency program or going unmatched altogether may also contribute to the observed discrepancies, but our study data do not make this distinction. Regardless, our research shows that a growing interest in neurology application does not guarantee increased representation in the workforce. More concerted efforts must be made to support underrepresented groups and create meaningful changes that positively influence an increasingly diverse patient population.

Given the general shortage of neurologists and the need for equitable patient care, institutions must bolster support for historically underrepresented individuals pursuing careers in neurology. Practitioners and trainees recognize that favorable experiences in neuroscience are crucial in residency recruitment.^28,30,32,37^ Studies have proposed that neurology departments collaborate with college neuroscience programs to offer research opportunities and foster early interest in clinical applications.^38^ The PARAdiGM program—which provides longitudinal mentorship, clinical exposure, research experience, and application assistance for underrepresented undergraduates—has shown success in supporting URiM trainees along their physician-scientist pathway.^39^ Participants in this program reported increased confidence and commitment to research as well as appreciation for a supportive community.^39^ Robust preclinical neuroscience courses and neurology clinical electives in medical school are also essential recruitment tools, as there is often a causal link between clinical exposure and a student's chosen specialty.^31,40-42^ Programs similar to Education in Pediatrics Across the Continuum provide medical students with longitudinal exposure to a field of interest which informs their career choices.^24,43^

The presence of a racially diverse physician leadership has further been identified as a significant factor in URiM students' decision to pursue specialties like neurology.^44^ URiM mentees who have a shared identity with their mentors report greater feelings of trust and support within the professional relationship.^44^ However, fewer than 10% of neurology residency program directors identify as Black or Hispanic.^45^ Thus, it is possible that URiM students may be less inclined to pursue a specialty in which there are limited physicians with whom they identify.

While medical schools and residencies must address the lack of representation among faculty and leadership, there are other solutions. Increased mentorship, regardless of physician race/ethnicity, creates a supportive environment for URiM trainees to explore their interest in neurology.^32,34,36,41^ Currently, programs such as the Student Interest Group in Neurology and the Medical Student Scholarship can improve access to networking and mentorship opportunities for URiM students.^41^ Initiatives that foster early exposure, mentorship, and academic support have been shown to increase the likelihood of underrepresented groups applying to their specialty of interest.^39,43^ Therefore, developing national research experiences and career immersion programs can further improve representation in neurology residency applicants and matriculants.

This study has several limitations. First, racial identity is self-reported and represents an inherent limitation. Racial identities differ substantially across geographical regions and through time and are open to sociopolitical interpretation. However, the use of self-reported racial and ethnic data has been validated for studies examining representation in medicine.^22-24^ Second, a portion of applicants self-identified as “other” or “unknown” race/ethnicity. It is unknown whether these individuals identified as multiracial or chose these selections for various reasons. Consequently, the representation of these individuals could not be properly assessed under a shared group. Third, racial/ethnic data were not available during the study period for international medical graduates and non-US citizens, who make up a large portion of those applying into neurology. The AAMC only provided these data for individuals who self-identified as US citizens or permanent residents before 2020. The absence of these data may have led to an underestimation of the representation of some racial identities. Fourth, our data did not specify whether individuals applied to neurology as a primary interest, nor did it include information on whether applicants ultimately matched to a different specialty. In addition, the authors were unable to identify those who re-applied to neurology because the applicant database did not distinguish first-time applicants from re-applicants. Fifth, the representational trends are presented in aggregate and do not necessarily represent the racial and gender diversity in specific neurology programs or geographical regions. The use of region-specific data would be useful in assessing geospatial differences in URiM representation in neurology. Sixth, the study period ends during the 2019–2020 residency application cycle, before the increase in the virtual interview format that resulted from the coronavirus disease 2019 pandemic. This drastic shift in format altered barriers that many face during an application cycle and potentially affected the representation of applicants and matriculants. Future research into these topics would further examine the reasons for underrepresentation and reduced matriculation in neurology residency.

In summary, this research highlights significant disparities in the representation of racial and ethnic minorities among neurology residency applicants and matriculants from the academic years of 2007–2008 through 2019–2020. Neurology applicant representation has increased during this 13-year study period, but changes in matriculant representation have not matched this pace. While there has been improvement in applicant diversity during this time, the representation of URiM matriculants in neurology residency programs has seen minimal change. Black men experienced a significant decline in representation from the applicant to matriculant stage. Meanwhile, Black women remained underrepresented as applicants and matriculants with no statistical improvement in representation. Disparities in neurology representation have broader implications when managing the multifaceted needs of an increasingly diverse patient. With the current workforce deficit, it is essential to implement strategies that attract diverse talent in neurology and support trainees through their professional development.

## Glossary

AAMC: Association of American Medical Colleges
AI/AN: American Indian/American Native
IQR: interquartile range
NHoPI: Native Hawaiian or Other Pacific Islander
PPIF: personal/professional identity formation
RQ: representation quotient
URiM: underrepresented in medicine
USMG: US medical school graduate
